# Genomic and functional analysis of the mucinolytic species *Clostridium celatum*, *Clostridium tertium*, and *Paraclostridium bifermentans*

**DOI:** 10.3389/fmicb.2024.1359726

**Published:** 2024-03-06

**Authors:** Francesco Candeliere, Eliana Musmeci, Laura Sola, Alberto Amaretti, Stefano Raimondi, Maddalena Rossi

**Affiliations:** ^1^Department of Life Sciences, University of Modena and Reggio Emilia, Modena, Italy; ^2^Department of Civil, Chemical, Environmental and Material Engineering (DICAM), Alma Mater Studiorum University of Bologna, Bologna, Italy; ^3^Biogest Siteia, University of Modena and Reggio Emilia, Reggio Emilia, Italy

**Keywords:** mucin, *Clostridium celatum*, *Clostridium tertium*, *Paraclostridium bifermentans*, functional genomics, human gut microbiota

## Abstract

Mucins are large glycoproteins whose degradation requires the expression of several glycosil hydrolases to catalyze the cleavage of the oligosaccharide chains and release monosaccharides that can be assimilated. In this study, we present a characterization on the strains *Clostridium celatum* WC0700, *Clostridium tertium* WC0709, and *Paraclostridium bifermentans* WC0705. These three strains were previously isolated from enrichment cultures on mucin of fecal samples from healthy subjects and can use mucin as sole carbon and nitrogen source. Genome analysis and *in vitro* functional analysis of these strains elucidated their physiological and biochemical features. *C. celatum* WC0700 harbored the highest number of glycosyl hydrolases specific for mucin degradation, while *P. bifermentans* WC0705 had the least. These predicted differences were confirmed growing the strains on 5 mucin-decorating monosaccharides (L-fucose, N-Acetylneuraminic acid, galactose, N-acetylgalactosamine, and N-acetylglucosamine) as only source of carbon. Fermenting mucin, they all produced formic, acetic, propionic, butyric, isovaleric, and lactic acids, and ethanol; acetic acid was the main primary metabolite. Further catabolic capabilities were investigated, as well as antibiotic susceptibility, biofilm formation, tolerance to oxygen and temperature. The potential pathogenicity of the strains was evaluated through *in silico* research of virulence factors. The merge between comparative and functional genomics and biochemical/physiological characterization provided a comprehensive view of these mucin degraders, reassuring on the safety of these species and leaving ample scope for deeper investigations on the relationship with the host and for assessing if some relevant health-promoting effect could be ascribed to these SCFA producing species.

## 1 Introduction

The epithelia are covered by mucus, that protects from luminal challenges and microbial infiltration. In the colon, mucus lubricates luminal contents and acts as a physical barrier against microorganisms, digestive enzymes and acids, digested food particles, microbial by-products, and food-associated toxins (Johansson et al., 2013). In the colon, mucus is produced by goblet cells and organized in two gel layers composed mainly of a net-like structure of MUC2 mucin (Johansson et al., 2011; Bansil and Turner, 2018). The outer mucus layer that interacts with the gut content is loose and inhabited by a specific microbiota, whereas the inner layer is much denser, more compact, tightly adhered to the epithelium, and prevents gut microbes from contacting the colon surface and causing inflammation (Berberolli et al., 2024). Impairment of the mucus barrier can allow bacteria to directly contact colorectal epithelial cells, triggering an inflammatory response (Fekete and Buret, 2023). A healthy mucus layer offers a proper environment that favors mutualistic bacteria and restricts growth of pathogens and pathobionts, providing energy, carbon, and nitrogen sources for herein residing human gut microbes (Arike and Hansson, 2016; Fekete and Buret, 2023; Berberolli et al., 2024).

Mucus is primarily composed of hydrophilic, branched glycoproteins, with mucins being the main components, that play a crucial role in the interaction with the external environment and have a significant impact on its viscosity. Mucins are large glycoproteins composed by repeating amino acid motifs with high abundance of proline, threonine, and serine, and heterogeneous *O*-linked glycans, the latter making up to 80% of the weight of MUC2 mucin (Luis and Hansson, 2023). The sugar moieties that compose the oligosaccharides decorating mucins are galactose, N-acetylglucosamine (GlcNAc), N-acetylgalactosamine (GalNAc), fucose, and N-Acetylneuraminic acid (Neu5Ac), with smaller amounts of mannose. The GalNAc is the first monosaccharide of the glycan chain, linked by an *O*-glycosidic bond to serine or threonine residues.

A rich commensal microbial community colonizes the outer mucus layer of the gut, being spatially organized along the length of the intestine as well as from the luminal to mucosal axis. Mucins provide carbon, nitrogen, and energy sources, and select specific microbes able to utilize these substrates (Arike and Hansson, 2016; Berberolli et al., 2024). Moreover, *O-*glycan chains of mucins trap microorganisms and serve as attachment sites for bacteria, preventing them from reaching the epithelial cells (Bergstrom et al., 2020). Interaction between host and microorganisms finely regulate both microbial and host physiology, promoting host tolerance toward commensal and pathogenic microbiota (Bergstrom and Xia, 2022). Many enteropathogenic bacteria have developed mechanisms to breach the mucus barrier, e.g., through flagella-driven propulsion (Sheikh et al., 2022). Dysbiosis is associated with mucus barrier disfunctions, bacterial penetration of the inner mucus layer, and reduction of core mucus components, all together resulting in the onset of inflammation and in pathogenesis of several diseases (Fang et al., 2021; Juge, 2022). For instance, a thinner mucus layer and an increased penetration of bacteria into the inner mucus layer are associated with intestinal inflammation and IBD incidence (Fekete and Buret, 2023).

Main mucus degrading bacteria belong to the taxa *Akkermansia muciniphila*, *Bacteroides, Bifidobacterium, Ruminococcus, Clostridium, Paraclostridium*, and *Prevotella* (Tailford et al., 2015; Crouch et al., 2020). Furthermore, we recently isolated strains of *Clostridium tertium*, *Clostridium celatum*, and *Paraclostridium bifermentans* from fecal sample enrichments of healthy subjects using mucin as the sole carbon and nitrogen source (Raimondi et al., 2021b), suggesting that a better understanding of the physiology of other mucin degrading bacteria, that may affect the bidirectional communication between microbiome residing in the mucus layer and the host, is required.

The ability of bacteria to degrade mucins depends on the expression of a number of glycosil hydrolases that catalyze the cleavage of the oligosaccharide chains, releasing monosaccharides and oligosaccharides which can be assimilated (Corfield et al., 1992; Luis and Hansson, 2023). The first step of mucin degradation is the hydrolysis of peripheral residues such as Neu5Ac and fucose by the exo-acting GHs neuraminidases/sialidases (GH33) and fucosidases (GH29 and GH95). Following the removal of terminal sugars, the complete hydrolysis of the oligosaccharides requires the activity of galactosidases (GH2, GH35, GH42, GH98), N-acetylhexosaminidases (GH20, GH84, GH85, GH89), and finally α-N-acetylgalactosaminidases (GH101, GH129) that cleave the linkage with the protein backbone, further susceptible to bacteria protease attack.

In this study, the physiological and biochemical features of the mucin degraders *Clostridium celatum* WC0700, *Clostridium tertium* WC0709, and *Paraclostridium bifermentans* WC0705 were investigated. A recent study focused on taxonomy and phylogeny of intestinal clostridia suggested a revision of the current classification of these species, assigning *C. celatum* and *C. tertium* to genus G14 and *P. bifermentans* to the closely related genus G15, both clustered in the main evolutive clade C3 that remotely diverged from other Clostridia (Candeliere et al., 2023). The genomes of *Clostridium celatum* WC0700, *Clostridium tertium* WC0709, and *Paraclostridium bifermentans* WC0705 were scanned to identify the genes encoding the enzymes responsible of mucin degradation, metabolism of carbohydrates, bases, amino acids, and vitamins, antibiotic resistance, virulence, bacteriocin biosynthesis, as well as phages, transposons, and insertion sequences. The findings from comparative and functional genomics were integrated with biochemical data to provide a comprehensive insight into these less-studied human gut mucin degraders. Safety assessment and investigation of technological properties have been carried out in the perspective of handling the strains to obtain alive microbial biomass for deeper investigations, such as *in vitro* and *in vivo* characterization of immunoregulatory properties and, at best, potential biotechnological exploitation.

## 2 Materials and methods

### 2.1 Strains, media, and culture conditions

*Clostridium celatum* WC0700, *Clostridium tertium* WC0709, and *Paraclostridium bifermentans* WC0705 were isolated from fecal cultures enriched on mucin, in order to identify mucin-degrading bacteria (Raimondi et al., 2021b). The strains were cultured in mucin medium (MM) (Raimondi et al., 2021b) in anaerobic conditions into butyl-rubber stoppered tubes to determine the extent of grow in terms of OD_600_ units.

The ability of the three strains to grow fermenting the single monomeric units that compose mucin oligosaccharides was assessed on basal M17 broth (BD Difco, Sparks, USA) supplemented of D-Glucose, D-Galactose (Sigma, Darmstadt, Germany), L-Fucose, Neu5Ac, GlcNAc, or GalNAc (Carbosynth, Staad, Switzerland), at the final concentration of 20 mM. M17 medium without any carbon source was used as negative control. The pH of the media was adjusted to 6.9–7.2 with 1 M HCl, then 12 mL of medium were dispensed into butyl-rubber stoppered tubes and sterilized. To obtain inocula where the carbon source was depleted, *C. celatum* WC0700, *C. tertium* WC0709, and *P. bifermentans* WC0705 were grown for two consecutive steps on M17 medium with lactose (2.9 mM) at 37°C for 48 h. These cultures were used to inoculate (10% v/v) tubes containing M17 supplemented with the diverse carbohydrates and the negative controls. For each medium, three subcultures were carried out, each one in triplicate, incubating the cultures at 37°C for 48 h. At the end of each step, OD_600_ was measured. In order to assess whether the fermentation pathways produced hydrogen, qualitative analysis of the headspace was carried out with a μGC 3000A (Agilent Technologies, Milano, Italy) under the following conditions: injector temperature 90°C; column temperature 60°C; sampling time 20 s; injection time 50 ms; column pressure 25 psi; run time 45 s and nitrogen as carrier gas.

### 2.2 Chemical analysis

Organic acids of the culture supernatants were quantified by HPLC with refractive index detector (1,200 System, Agilent Technologies, Waldbronn, Germany) and Aminex HPX-87 H ion exclusion column. Isocratic elution was carried out at 60°C with 0.8 mL min^−1^ of 5 mM H_2_SO_4_ (Amaretti et al., 2013).

### 2.3 Biochemical characterization

*C. celatum* WC0700, *C. tertium* WC0709, and *P. bifermentans* WC0705 were tested for the fermentation of 49 carbohydrates and other carbohydrate derived molecules using API 50 CH test strips (bioMerieux, Marcy, l’Etoile, France). Bacterial biomass from the surface of M17-glucose agar plates (BD Difco, Sparks, USA) was harvested and resuspended in API 50 CHL medium at 2 McFarland units. The suspension was used to inoculate the strips, according to manufacturer instructions. Growth occurred in the anaerobic chamber for 48 h at 37°C.

### 2.4 Biofilm production

The ability of *C. celatum* WC0700, *C. tertium* WC0709, and *P. bifermentans* WC0705 to form biofilm and to adhere to a mucin covered surface was tested. Purified mucin type III (Sigma-Aldrich, Darmstadt, Germany) was dissolved in phosphate-buffered saline (PBS) pH 7.4 to a final concentration of 1 mg/mL. Each well of 96-well polystyrene microtiter plates was loaded with 200 μL of the mucin suspension; the plates were maintained overnight at 4°C. Unbound mucin was removed washing wells with PBS, according to Sadiq et al. (2021). Cultures grown anaerobically in M17-glucose for 48 h were diluted (10% v/v) in fresh M17-glucose medium and seeded in mucin-coated and uncoated wells. Plates were incubated in anaerobiosis for 48 h at 37°C and biofilm formation was assayed by crystal violet (CV) staining according to Raimondi et al. (2019). Briefly, unattached cells were discarded, and each well was washed three times with PBS. Biofilm was stained with CV solution (0.1%) for 15 min, then excess of staining was removed and wells were washed three times with PBS. De-staining solution (80% v/v ethanol, and 20% v/v acetone) was added to release the stain, and biofilm was quantified by measuring OD_570_. Specific biofilm formation (SBF) index was calculated as the ratio between CV absorbance at 570 nm and culture’s turbidity at 620 nm, setting a threshold of 1. The strains *Escherichia coli* 03.73 and *Klebsiella pneumoniae* 11.71 (Raimondi et al., 2019; Amaretti et al., 2020) were used as positive and negative controls, respectively.

### 2.5 Tolerance to oxygen and temperature

Tolerance to oxygen and to high temperatures of *C. celatum* WC0700, *C. tertium* WC0709, *P. bifermentans* WC0705 were tested on 48 h cultures grown in M17-glucose broth at 37°C. Portions of the same culture were: (I) exposed to air in a 10x volume baffled flask maintained at 37°C for 1 h in an orbital shaker (180 rpm); (II) heated at 80°C for 30 min; (III) exposed to air, then heated at 80°C for 30 min (I + II). After each treatment, serial dilutions in M17-glucose were made in anaerobic tubes, that were incubated at 37°C for 48 h. The Most Probable Number (MPN) method was used to estimate microbial population size, using untreated cultures as controls.

### 2.6 Antibiotic susceptibility

The susceptibility to antibiotics of *C. celatum* WC0700, *C. tertium* WC0709, and *P. bifermentans* WC0705 was assayed with broth microdilution method according to the International Standard Organization (ISO 20776-1:2019), Cultures grown in M17-glucose medium for 48 h at 37°C were diluted in fresh M17-glucose broth to obtain a final concentration of 5 × 10^5^ cfu/mL. Ampicillin, gentamicin, and chloramphenicol were tested at doubling dilutions, from 0.06 to 64 mg/L; penicillin G and tetracycline from 0.015 to 16 mg/L. The antibiotic susceptibility was determined after 48 h of incubation at 37°C in anaerobiosis. MICs were defined according to the European Committee on Antimicrobial Susceptibility Testing breakpoints (EUCAST)^1^ for Gram-positive anaerobes, except for tetracycline and penicillin G, for which breakpoints were defined according to Clinical and Laboratory Standards Institute (CLSI)^2^. No defined breakpoints were available for gentamicin in the two databases. Indicatively, the gentamicin breakpoint of 16 was chosen, according to Wei et al. (2020), referring to the CLSI-M100-S23:2019 database.

### 2.7 Statistical analysis

The data reported are the means of at least three independent experiments, each carried out in triplicate. The statistical significance was analyzed with *t*-test (*p* < 0.05). Statistical analysis was performed using GraphPad Prism 7 software (GraphPad, San Diego, CA, United States). Statistical differences were assessed by one-way ANOVA and Bonferroni multiple-comparison *post hoc* tests. Differences were considered significant at *p* < 0.05.

### 2.8 Genome extraction and sequencing

Bacterial cells of *C. celatum* WC0700, *C. tertium* WC0709, and *P. bifermentans* WC0705 grown in MM at 37°C for 48 h under strictly anaerobic conditions was collected by centrifugation for 10 min at 12,000 g. The genomic DNA was extracted with DNeasy Blood & Tissue kit (Qiagen GmbH, Düsseldorf, Germany). Before DNA purification, the pre-treatment for Gram-positive bacteria was performed following manufacturer’s specific, with some modifications: longer incubation times (2 h at 37°C and 1 h at 56°C) and twofold the volume of enzymatic lysis buffer, proteinase K and Buffer AL. The quality of the DNA was checked with a Nanodrop spectrophotometer, and the concentration was quantified with a Qubit 3.0 fluorimeter (Thermo Fisher Scientific, Waltham, MA, USA). The samples were sequenced with Illumina NovaSeq 6000 by Eurofins Genomics (Ebersberg Germany). For each sample, 150-bp paired-end reads were obtained. Assembled genomes of *C. celatum* WC0700, *C. tertium* WC0709, and *P. bifermentans* WC0705 were submitted to NCBI with the accession number, PRJNA781812, PRJNA737738, PRJNA781829, respectively. Strains WC0700 and WC0705 were sequenced for this work, while WC0709 was published previously (Musmeci et al., 2021).

### 2.9 Genome assembly

The raw reads were checked for quality with FastQC v0.11.8 (Andrews, 2010). To trim Illumina adapters and remove reads with a low quality score, Cutadapt v1.16 was used with the following parameters: overlap = 15, minimum length = 30 and quality-cutoff = 20 (Martin, 2011). The trimmed reads were assembled with SPAdes v3.13.0 (−-careful --cov-cutoff auto -k auto) (Bankevich et al., 2012). The quality of the assemblies was evaluated using QUAST 5.0.2 (Gurevich et al., 2013). Trimming, quality checking, and assembly were performed on Galaxy platform^3^ (Afgan et al., 2018). The taxonomy was assessed with TypeMat of Microbial Genomes Atlas (MIGA; Rodriguez-R et al., 2020). The completeness of the assemblies was verified with CheckM 1.0.8 (Parks et al., 2015).

### 2.10 Genomes annotation and functional characterization

The annotation was carried out with the tool Prokka v1.14.5 (Seemann, 2014) and the hypothetical proteins were further annotated with eggnog-mapper v2 (Huerta-Cepas et al., 2019; Cantalapiedra et al., 2021) and InterPro Scan 5.60–92.0 (Jones et al., 2014; Blum et al., 2021). KEGG tools, BlastKOALA and MAPPER (Kanehisa et al., 2016; Kanehisa and Sato, 2020), and the tool gapseq (Zimmermann et al., 2021) were used to predict functional properties of the genomes. The dbCAN2 metaserver was used for the annotation of Carbohydrate Active Enzyme (CAZymes) (Zhang et al., 2018), to identify the genes encoding glycoside hydrolases (GH), carbohydrate esterases (CE), glycosyl transferases (GT), carbohydrate-binding modules (CBM), and polysaccharide lyases (PL). Only the CAZymes annotated by two out of the three tools (HMMER, Hotpep, and Diamond) used by dbCAN2 were considered. The putative glycoside hydrolases involved in the mucin degradation (GH2, GH20, GH29, GH33, GH35, GH42, GH84, GH85, GH89, GH95, GH98, GH101, and GH129) were searched. Enzymes involved in mucin monomers (D-Galactose, L-Fucose, Neu5Ac, GlcNAc, and GalNAc) utilization were also searched through BLAST using the sequences identified by Ravcheev and Thiele (2017) as references.

In order to investigate the presence of an adaptive immunity systems, CRISPRs and Cas genes were searched using CRISPRCasFinder (Couvin et al., 2018), with default settings and subtype clustering of Cas genes. The presence of prophage sequences was analyzed with PHASTER (Arndt et al., 2016). To assess the potential of these strains to share genetic resistance determinants, the identification of antibiotic resistance genes was carried out using the web tool RGI (Resistance Gene Identifier) of CARD (Comprehensive Antibiotic Resistance Database), processing the contigs file for “Perfect, Strict and Loose hits” (Alcock et al., 2020). Insertion sequences were identified with ISFinder (Siguier et al., 2006). BAGEL 4 server was used to search bacteriocins genes (Van Heel et al., 2018).

The presence of putative virulence factors was investigated with Virulence factor database (VFDB; Liu et al., 2022) to assess the pathogenic potential of these strains. This tool allowed also to compare the pathogenicity of the three strains against *C. perfringens* and *Clostridioides difficile*. Pathogenicity and virulence factors were also investigated with PathogenFinder v1.1 (Cosentino et al., 2013).

### 2.11 Comparative genomics

The available genomes of *C. celatum*, *C. tertium*, and *P. bifermentans* strains were downloaded from GenBank (Supplementary Table S1) on 16 January 2023. Metagenome-assembled genomes (MAG) and genomes with a contamination >5% after quality check with CheckM were excluded. As a whole, comparative genomics investigated 4 genomes of *C. celatum*, 23 of *P. bifermentans,* and 12 of *C. tertium.* Average Nucleotide Identity (ANI) and digital-DNA/DNA hybridization (dDDH) were calculated using the ANI Matrix web tool (Rodriguez-R and Konstantinidis, 2016) and Genome-to-Genome Distance Calculator GGDC 2.1,^4^ with the thresholds for species demarcation of 95% for ANI and 70% for dDDH (Richter and Rossello, 2009). Panaroo was utilized to calculate the pangenome and to define core and accessory genes, utilizing Prokka annotation files (Tonkin-Hill et al., 2020). Default settings were applied to Panaroo’s runs (--clean-mode strict, --core_threshold 0.98, --len_dif_percent 0.98). Genes in the pangenome were categorized as core if present in all the strains, soft core if present in 95–99% of strains, shell genes in 95–15% of strains, cloud genes in less than 15% of strains.

## 3 Results

### 3.1 Taxonomic attribution

The taxonomy of the strains WC0700, WC0709, and WC0705, which were initially identified as *Clostridium disporicum, Clostridium tertium,* and *Paraclostridium benzoelyticum* based on partial 16S rRNA gene sequencing (Raimondi et al., 2021b), was re-evaluated using TypeMat. The proper taxonomy of the isolates was *Clostridium celatum* WC0700, *C. tertium* WC0709, and *Paraclostridium bifermentans* WC0705. To confirm the taxonomic assignment, digital DNA–DNA hybridization (dDDH) and average nucleotide identity (ANI) values were calculated comparing each isolate with the relative reference strain available (Table 1). All the isolates were over the thresholds for species demarcation (70 and 95% for dDDH and ANI, respectively; Richter and Rossello, 2009).

**Table 1:** The digital DNA–DNA hybridization (dDDH) and average nucleotide identity (ANI) values between *C. celatum* WC0700, *C. tertium* WC0709, and *P. bifermentans* WC0705 and the related type strains.

Isolate	Reference strain	dDDH (%)	ANI (%)
*Clostridium celatum* WC0700	*C. celatum* DSM 1785^T^	99.2	99.92
*Clostridium tertium* WC0709	*C. tertium* DSM 2485^T^	88.7	98.70
*Paraclostridium bifermentans* WC0705	*P. bifermentans* ATCC 638^T^	89.6	98.94

### 3.2 Genome features

In the 3 genomes, whose main features are presented in Supplementary Table S2, a high number of proteins were annotated as functionally uncharacterized hypothetical proteins using Prokka (41.9–44.8%). A deeper annotation carried out with eggnog-mapper and InterProScan allowed to reduce the number of hypothetical proteins to 12.7–15.7%.

None of the three strains possessed CRISPR-Cas systems (Stern et al., 2012). On the other hand, a number of genes encoding restriction systems were identified in the genomes of *C. celatum* WC0700 (22)*, C. tertium* WC0709 (8), and *P. benzoelyticum* WC0705 (12). Only in the genome of *C. celatum* WC0700 a prophage sequence was identified. Seven ISs were identified in *C. celatum* WC0700, belonging to the families IS1595, IS6, and IS200/IS605. They presented similarities with others from *Campylobacter jejuni* and *C. disporicum* (IS1595), *C. tetani* (IS6), and *C. perfringens* (IS200/IS605). *P. bifermentans* WC0705 encompassed two ISs sequences of the families IS607 (*C. botulinum*) and IS1182 (*Bacillus cytotoxicus*). ISs of the families IS3 and IS200/IS605 were found in *C. tertium* WC0709 and presented similarities with the counterparts of *C. beijerinckii* and *Staphylococcus epidermidis,* respectively.

The pangenome of the three species was also analyzed, retrieving high quality genomes from GenBank (Supplementary Table S2; Supplementary Figure S1A). Given the low number of available genomes, for *C. celatum* it was not possible to calculate the γ value to establish if it was a closed or open pangenome (Supplementary Figure S1B). According to Heap’s law, the pangenomes of *P. bifermentans* and *C. tertium* were considered open, with a γ value of 0.44 and 0.53, respectively, (Tettelin et al., 2008).

### 3.3 Functional analysis

#### 3.3.1 Mucin hydrolysis

The genome of *C. celatum* WC0700, *C. tertium* WC0709, and *P. bifermentans* WC0705 was interrogated with CAZY database, in order to predict the presence of enzymes that degrade, modify, or create glycosidic bonds (Table 2). The GHs putatively involved in the hydrolysis of mucin are reported in Table 3. GH35, GH42, GH85, GH98, and GH129 were not identified in any of the isolated strain. *C. disporicum* WC0700 exhibited the most diverse and extensive collection of GHs specifically targeting mucins’ *O*-glycans, most of which possessing signal peptides. Similarly, *C. tertium* WC0709 possessed a substantial array of GHs but had fewer genes and lacked GH89. *P. bifermentas* had the fewest GHs, featuring only one galactosidase (GH2) and one hexosaminidase (GH20).

**Table 2:** CAZymes identified in the genomes of *C. celatum* WC0700, *C. tertium* WC0709, and *P. bifermentans* WC0705 with at least two out the three dbCAN tools.

	*C. celatum* WC0700	*C. tertium* WC0709	*P. bifermentans* WC0705
Glycoside Hydrolase (GH)	64 (28)	78 (15)	24 (1)
Carbohydrate esterase (CE)	9 (2)	10 (4)	9 (4)
Glycosyl transferase (GT)	22 (0)	30 (0)	16 (0)
Carbohydrate-binding module (CBM)	27 (19)	16 (9)	3 (1)
Polysaccharide lyase (PL)	0	4 (0)	0
Total	122	138	52

For each Cazy class, the number of genes identified is reported, with those harboring a signal peptide in brackets.

**Table 3:** Glycosyl hydrolases (GHs) putatively involved in mucin hydrolysis identified in the genomes of *C. celatum* WC0700, *C. tertium* WC0709, and *P. bifermentans* WC0705 with at least two out the three dbCAN tools.

Putative CAZY activity	GH domain	*C. celatum* WC 0700	*C. tertium* WC 0709	*P. bifermentas* WC0705
Exo-α-sialidase	GH33	2 (2)	1 (1)	
Fucosidase	GH29	1 (1)		
Exo-α-fucosidase	GH95	2 (2)		
Exo-β-galactosidase/glucuronidase	GH2	5 (3)	4 (1)	1 (0)
Exo-α/β-N-acetylglucosaminidase	GH20	4 (3)	4 (2)	1 (0)	GH84	5 (4)	1 (1)		GH89	1 (1)		
Endo-α/β-N-acetylgalactosaminidase	GH101	5 (4)	1 (1)	

For each GH, the number of genes identified is reported, with those harboring a signal peptide in brackets. GH35, GH42, GH85, GH98, and GH129 were not identified.

**Figure 1:**
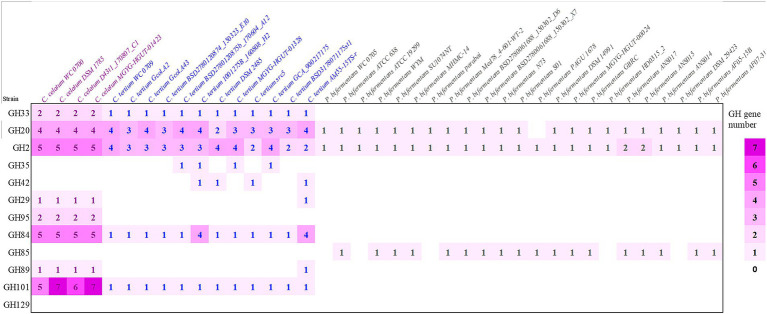
Predicted genes encoding enzymes of the GH families involved in mucin O-glycan hydrolysis in strains belonging to the species *Clostridium celatum*, *Clostridium tertium, and Paraclostridium bifermentans*. The heat map shows the gene number of each GH in all the examined genomes: sialidase (GH33), fucosidases (GH29, GH95), galactosidases (GH2, GH35, GH42), N-acetylhexosaminidases (GH20, GH84, GH85, GH89) and α-N-acetylgalactosaminidases (GH101, GH129).

The presence of genes encoding mucin *O*-glycans degrading enzymes was also evaluated in the set of strains belonging to the *C. celatum*, *C. tertium*, and *P. bifermentans* available in GenBank repository (Figure 1).

Taking into account the other genomes retrieved from GenBank, *C. celatum* harbored 25 to 27 GHs involved in mucin degradation: 2 sialidases (GH33); 3 fucosidases (2 GH95 and 1 GH29); 5 galactosidases (GH2); 10 hexosaminidases belonging to the families GH20 (4), GH84 (5), and GH89 (1); and 5 to 7 GH101 endo-α-*N*-acetylgalactosaminidases.

The genomes *C. tertium* harbored 8 to 15 GH genes including a sialidase (GH33) and a number of β-galactosidases. For 5 out of 12 *C. tertium* strains, β-galactosidase activity seemed attributed only to GH2 enzymes, lacking both GH35 and GH42 genes. Generally, the *C. tertium* strains encoding a GH35 did not harbor a GH42 gene, and vice versa. Fucosidases ascribed to families GH29 or GH95 were not detected, except for *C. tertium* AM55-15TS-r. This strain was also the sole *C. tertium* with predicted α-N-acetylglucosaminidase activity (1 GH89 gene). All the strains harbored 2–4 genes encoding an exo-β-N-acetylglucosaminidases GH20 and one gene for endo-α-*N*-acetylgalactosaminidase (GH101).

*Paraclostridium bifermentans* encoded only 2 to 4 GH genes potentially involved in mucin degradation. All the strains possessed one or two β-galactosidase (GH2) genes. Most of the strains also encoded an exo-α/β-N-acetylglucosaminidase (GH20) and a mannosyl-glycoprotein endo-β-N-acetylglucosaminidase (GH85).

#### 3.3.2 Metabolism of carbohydrates

**Figure 2:**
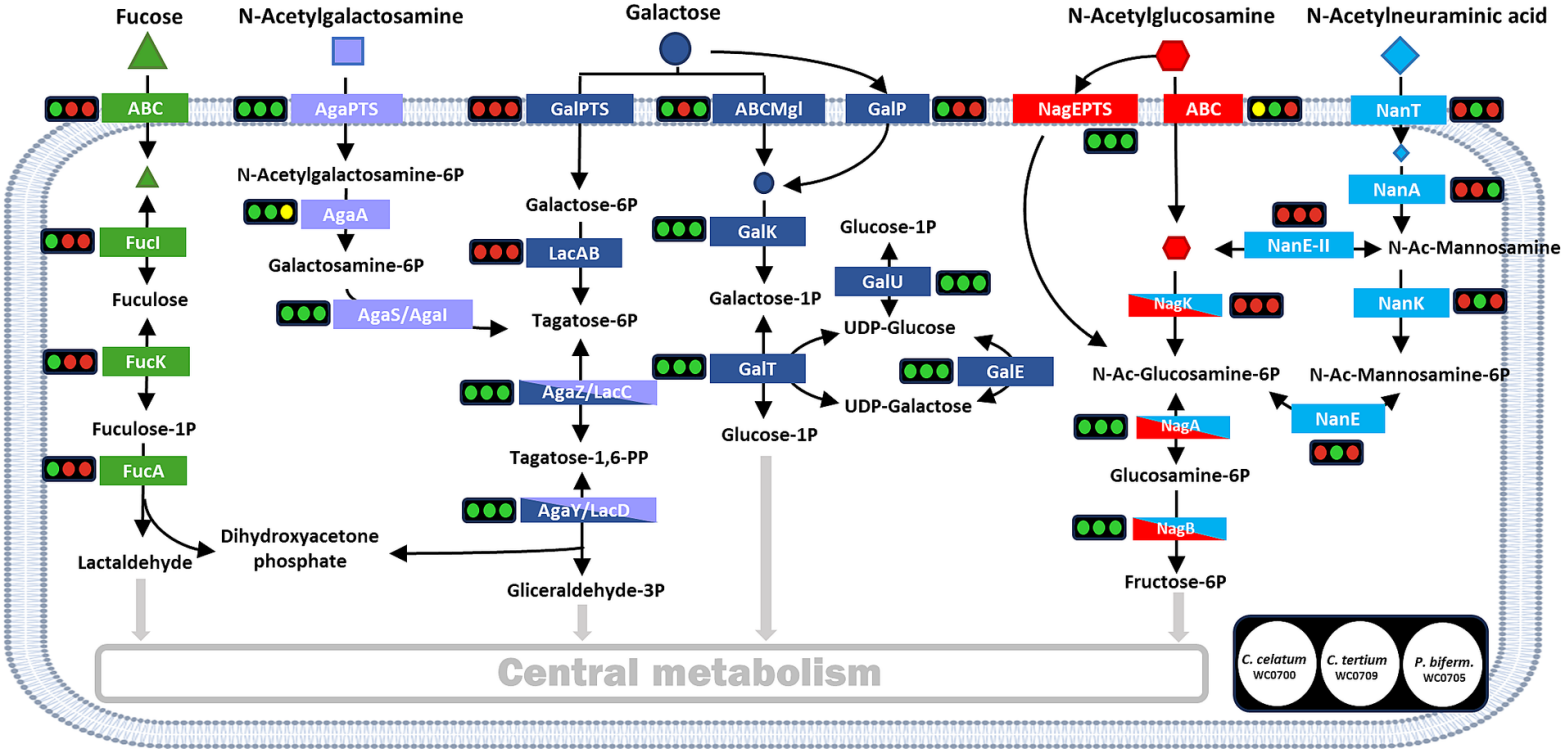
Reconstructed pathways for the utilization of mucin monomers. For each enzyme or metabolic block, the circles indicate the presence (green) or absence (red) in the genome of *C. celatum* WC0700, *C. tertium* WC0709, and *P. bifermentans* WC0705, in this order. Yellow circles indicate uncertainty, due to the lack of subunits or components. Figure adapted from Ravcheev and Thiele (2017).

The genome of *C. celatum* WC0700, *C. tertium* WC0709, and *P. bifermentans* WC0705 was annotated using KEGG, in order to reconstruct the metabolic potential of the strains (Supplementary Spreadsheet 1) and, in particular, their ability to utilize mucin (Figure 2). Specific functions related to mucin fermentation were searched with BLAST.

The genome of *C. celatum* WC0700, *C. tertium* WC0709, and *P. bifermentans* WC0705 harbored a variety of complete phosphotransferase systems (PTS) and ABC transporters (Supplementary Spreadsheet 1), putatively enabling the uptake of several simple sugars and oligosaccharides. Carbohydrates that could be transported into the bacterial strains include constitutive moieites of mucin *O*-glycans (galactose, fucose, GlcNAc, and GalNAc), in addition to other mono and oligosaccharides (glucose, fructose, mannose, ribose and xylose, aldouronates, maltose, sucrose, cellobiose, galactosamine, β-glucoside-methyl-galactoside, and raffinose, stachyose, and melibiose). *C. celatum* WC0700 was the only strain harboring galactose permease GalP, whereas *C. tertium* WC0709 was the sole strain where no galactose transporter was identified. Neu5Ac transporter NanT was identified only in *C. tertium* WC0709. Other Neu5Ac transporters were not identified in any of the strains.

The metabolic blocks (as defined by KEGG Module)^5^ of the pathways directing mucin-derived sugars (fucose, Gal, GalNAc, GlcNAc, and Neu5Ac) into the central carbon metabolism and enabling their fermentation were searched. Only *C. celatum* WC0700 harbored fucose isomerase (*fucI*), fuculokinase (*fucK*), and fuculose phosphate aldolase (*fucA*) to metabolize fucose into lactaldehyde, consistently with the presence of GH29 and GH95 fucosidases and an ABC-type fucose transport system in this specific strain. Galactose utilization could take place in all the strains through Leloir’s pathway, yielding glucose-1-phosphate and then glucose-6-phosphate. Tagatose-6-phosphate pathway, the alternative route for galactose catabolism yielding glyceraldehyde 3-phosphate, was complete in all the strains except for the absence of galactose-6P isomerase (*lacA*), the initial enzyme of this route (Figure 2).

The two metabolic blocks necessary to introduce GalNAc-6P into tagatose-6-phosphate pathway were found in *C. celatum* WC0700 and *C. tertium* WC0709. The first block, i.e., GalNAc-6P deacetylase encoded by *AgaA*, was not predicted in *P. bifermentans* WC0705. Since *nagA* and *AgaA* complement each other fulfilling the same function in *E. coli* (Hu et al., 2013), it remains uncertain whether *nagA* could provide for the absence of *AgaA* in *P. bifermentans*.

In the three strains, GlcNAc-6P could be deacetylated by the deacetylase encoded by *nagA* and channeled to fructose-6P by the enzymes encoded by *nagA* and *nagB*. Even though both *C. celatum* WC0700 and *C. tertium* WC0709 were equipped with GH33 sialidases, only *C. tertium* encoded the enzymes involved in Neu5Ac utilization, i.e., the *nanK* kinase and the *nanE* epimerase responsible for the conversion of *N*-acetylmannosamine-6P into *N*-acetylglucosamine-6P (Figure 2).

With regards to other catabolic routes, the pathways for the degradation of ascorbate, glucuronate, and galacturonate were incomplete or missing. Glycogen/starch degradation was predicted in *C. tertium* WC0709 and *C. celatum* WC0700, while *P. bifermentans* WC0705 lacked 4-α-glucotransferase (*malQ*) or pullulanase (*pulA*) in the degradation pathway (Supplementary Spreadsheet 1).

*C. celatum* WC0700, *C. tertium* WC0709, and *P. bifermentans* WC0705 shared the pathways for Embden-Meyerhof glycolysis, the non-oxidative phase of the pentose phosphate pathway, and the conversion of pyruvate to acetyl-CoA, while Entner-Douduroff glycolysis and the tricarboxylic and glyoxylate cycles were extensively incomplete (Supplementary Spreadsheet 1). The genes encoding the enzymes involved in clostridial-type fermentations were specifically searched. The reconstructed scheme of biochemical reactions is reported in Supplementary Figure S2. The routes stemming from glycolisis and leading to formate, lactate, acetate, ethanol, butyrate, and butanol were complete in all the strains, except for three blocks channeling acetyl-CoA toward butyryl-CoA that were not found in *P. bifermentans* WC0705. The route leading to acetone was absent in all strains. Hydrogen:ferredoxin oxidoreductases (Fd, blue in Supplementary Figure S2), releasing H_2_ and regenerating ferredoxins, were predicted in all the strains but their role in specific metabolic reactions remains unclear.

The lactaldehyde pathway, in orange in Supplementary Figure S2, involved in channeling glycolysis toward propionate and propanol, was found to be mostly complete in the three strains. The only uncertainty regards propanediol utilization, since the gene *pduC* was missing in *C. tertium* WC0709 and it is unclear whether other *pdu* genes found in *C. celatum* WC0700 and *P. bifermentans* WC0705 may fulfill this function. All the strains lacked the gene *pct*, the first block of lactate pathway for propionate production, while *C. celatum* WC0700 and *C. tertium* WC0709 lacked also the gene *LcdA*.

#### 3.3.3 Metabolism of bases, amino acids, and vitamins

The three genomes shared complete ABC transporters for the uptake of oligopeptides, basic amino acids, the biogenic amines spermidine and putrescine, choline (referred to as osmopretectant transporter), nucleosides, and biotin. A complete transporter for D-methionine was found in *C. tertium* WC0709 and *C. celatum* WC0700 (Supplementary Spreadsheet 1).

The three genomes harbored all or most of the genes involved in the biosynthetic routes of purine and pyrimidine nucleotides and deoxy-ribonucleotides. The anabolic pathways of many amino acids were extensively incomplete in the three bacteria. The three genomes harbored all the genes necessary for the biosynthesis of lysine, *C. celatum* WC0700 and *C. tertium* WC0709 using the succinyl-DAP pathway and *P. bifermentans* WC0705 using the DAP aminotransferase one. The metabolic route branching from lysin biosynthesis and yielding homoserine and then threonine was complete only in *C. celatum* WC0700 and *C. tertium* WC0709. The pathway transforming homoserine into methionine was incomplete in the three genomes. The pathway of *de novo* biosynthesis of serine was interrupted in all the genomes, that, on the other hand, were all equipped with the genes necessary to transform serine into cysteine. The pathways yielding valine, leucine, and isoleucine from pyruvate were complete in *C. celatum* WC0700 and *C. tertium* WC0709, while no genes for the *de novo* biosynthesis of branched chain amino acids were identified in *P. bifermentans* WC0705. Likewise, the pathways for the transformation of glutamate into proline and arginine via ornithine were complete only in *C. celatum* WC0700 and *C. tertium* WC0709. Complete shikimate pathway was predicted in the three genomes, whereas the route of transformation of chorismate into phenylalanine and tyrosine was always incomplete and the one leading to tryptophan was always missing. A complete pathway for histidine degradation was identified only in *P. bifermentans* WC0705. Genes involved in the interconversions between glutamate, ornithine, arginine, spermidine, and putrescine, were identified in the three genomes. On the other hand, the metabolic modules channeling them to GABA, and finally toward succinyl-CoA for degradation were interrupted.

Ammonia lyases, transforming serine and threonine into pyruvate and 2-oxobutanoate were found in all the strains, ultimately yielding organic acids, including propionate. Furthermore, all the strains harbored genes encoding several amino acid aminotransferases, including the one priming the degradation of leucin toward the production of isovalerate (Supplementary Figure S2).

The biosynthetic pathways of vitamins and cofactors were incomplete for the most part, with a few exceptions. The genome of *P. bifermentans* WC0705 encoded a complete ABC transporter for cobalt and all the enzymes necessary for *de novo* anaerobic production of cobalamin. Incompleteness was observed in the module yielding cobyrinate a,c-diamide in *C. celatum* WC0700 and *C. tertium* WC0709. CoA production from panthotenate was predicted in all the genomes, nonetheless pathways synthetizing panthotenate were incomplete. *C. tertium* WC0709 harbored the R5P pathway for pyridoxal phosphate biosynthesis. *C. celatum* WC0700 and *P. bifermentans* WC0705 had the pathway for thiamine monophosphate salvage.

#### 3.3.4 Virulence

The pathogenicity of *C. celatum* WC0700, *C. tertium* WC0709, and *P. bifermentans* WC0705 was investigated and compared with two well-known and closely related bacterial pathogens, *C. difficile* (unpublished results) and *C. perfringens* (Candeliere et al., 2023). The VFDB tool identified in all the isolates the genes *fbpA*, encoding fibronectin-binding protein, *groEL*, and two genes encoding hemolysins, one belonging to family III, the other potentially exhibiting both hemolytic and methyltransferase activity (TlyA family RNA methyltransferase) (Table 4; Supplementary Figure S3). These proteins were also encoded by both *C. difficile* and *C. perfringens*. Notably, homologs of four different *C. perfringens* hemolysins were identified in *C. tertium* WC0709. The gene encoding neuraminidase *NagH* (mu-toxin) was found in *C. celatum* WC0700 and *C. tertium* WC0709. The former also had the genes encoding neuraminidases *NagI* and *NagK*, and the sialidase *NanJ*. It is worth noting that, in this case, we do not consider neuraminidases and sialidases as toxins; instead, their presence is justified by their role to facilitate bacterial growth on mucin. *P. bifermentans* WC0705 harbored the genes encoding alpha-toxin *PLC*, collagenase *ColA* and perfringolysin *PfoA*, virulence determinants of *C. prefringens*.

**Table 4:** Virulence factors identified by VFDB tool.

Virulence factors	Related genes	*C. celatum* WC0700	*C. tertium* WC0709	*P. bifermentans* WC0705	*C. difficile* 630	*C. perfringens* ATCC 13124
CD0873	CD0873				X	
CD2831	CD2831				X	
CD3246	CD3246				X	
CbpA	*cbpA*				X	
Cna	*cna*					
Cwp66	*cwp66*				X	
Cwp84	*cwp84*				X	
CwpV	*cwpV*				X	
Fibronectin-binding protein	*fbpA/fbp68*	X	X	X	X	X
GroEL	*groEL*	X	X	X	X	X
S-layer protein	*slpA*				X	
Zmp1	*zmp1*				X	
VirR/VirS two component system	*virR*					X
	*virS*					X
Alpha-clostripain	*cloSI*					X
Alpha-toxin	*plc*			X		X
Beta2 toxin	*cpb2*					
Botulinum neurotoxin (BoNT)	*atx*					
*C. novyi* alpha-toxin	*tcnA*					
*C. perfringens* enterotoxin (CPE)	*cpe*					
*Clostridium difficile* toxin (CDT)	*cdtA*				X	
	*cdtB*				X	
Hemolysin	Hemolysin		X			X
	Hemolysin	X	X	X	X	X
	Hemolysin	X	X	X	X	X
	Hemolysin		X			X
Kappa-toxin (collagenase)	*colA*			X		X
Mu-toxin (neuraminidase)	*nagH*	X	X			X
	*nagI*	X				X
	*nagJ*					X
	*nagK*	X				X
	*nagL*					
Perfringolysin O (theta-toxin/PFO)/botulinolysin/tetanolysin O	*pfoA*			X		X
Sialidase	*nanH*					X
	*nanI*					X
	*nanJ*	X				X
Tetanus toxin (TeTx)	*tetX*					
Toxin A	*toxA*				X	
Toxin B	*toxB*				X	

Genes present in *C. celatum* WC0700, *C. tertium* WC0709, *P. bifermentans* WC0705, *C. difficile* 630, and *C. perfringens* ATCC 13124 are reported.

According to PathogenFinder, *C. celatum* and *C. tertium* were classified as non-human pathogens, whereas *P. bifermentans* exhibited a moderate probability of being a human pathogen (0.69). None of the proteins from *C. tertium* had a homolog in the pathogenic protein families database, whereas *C. celatum* and *P. bifermentans* exhibited 1 and 11 matches with pathogenic proteins generally encoded by *C. difficile* (Supplementary Table S3).

The gene encoding UviB, a protein that in *C. perfringens* is involved in release of the bacteriocin BCN5 from the cell, was present in the genome of *P. bifermentans* WC0705, and similar proteins were identified also in the genomes of *C. celatum* WC0700 and *C. tertium* WC0709. Only the genome of *C. celatum* WC0700 harbored also the gene *bcn5* encoding the corresponding bacteriocin. *C. tertium* WC0709 encoded complete ABC transporters for lantibiotics.

### 3.4 Biochemical features and fermentation of *O*-glycan monomers and mucin

The metabolic capabilities of *C. celatum* WC0700, *C. tertium* WC0709, and *P. bifermentans* WC0705 were assessed using API 50 CH (Table 5). *C. tertium* WC0709 exhibited the widest range of substrates. *P. bifermentans* WC0705 was the sole strain growing on gluconate, and 2-ketogluconate, while only *C. celatum* WC0700 fermented D-trehalose and L-fucose.

**Table 5:** Fermentation of different substrates (API 50 CH test) by *C. celatum* WC0700, *C. tertium* WC0709, and *P. bifermentans* WC0705.

	Control	Glycerol	Erythritol	D-arabinose.	L-arabinose	D-ribose	D-xylose	L-xylose	D-adonitol	Methyl-β-D-xylopyranoside	D-galactose	D-glucose	D-fructose	D-mannose	L-sorbose	L-rhamnose	Dulcitol	Inositol	D-mannitol	D-sorbitol	Methyl-α-D-mannopyranoside	Methyl-α-D-glucopyranoside	N-acetylglucosamine	Amygdalin	Arbutin
*C. celatum* WC0700	−	−	−	−	−	+	−	−	−	−	+	+	+	+	−	−	−	−	−	−	−	−	+	+	−
*C. tertium* WC0709	−	+	−	−	−	+	+	−	+	−	+	+	+	+	+	−	−	−	+	+	−	−	+	+	+
*P. bifermentans* WC0705	−	+	−	−	−	+	−	−	+	−	+	+	+	+	−	−	−	−	+	+	−	−	+	−	−
	Esculin ferric citrate	Salicin	D-cellobiose	D-maltose	D-lactose	D-melibiose	D-saccharose	D-trehalose	Inulin	D-melezitose	D-raffinose	starch	Glycogen	Xylitol	Gentiobiose	D-turanose	D-lyxose	D-tagatose	D-fucose	L-fucose	D-arabitol	L-arabitol	Potassium gluconate	Potassium 2-ketogluconate	Potassium 5-ketogluconate
*C. celatum* WC0700	+	−	+	+	+	+	+	+	−	−	−	−	−	−	+	−	−	−	−	+	−	−	−	−	−
*C. tertium* WC0709	+	+	+	+	+	+	+	−	−	+	−	−	−	−	+	−	−	+	−	−	−	−	−	−	−
*P. bifermentans* WC0705	+	−	+	+	+	−	−	−	−	−	−	−	−	−	−	−	−	−	−	−	−	−	+	+	−

**Figure 3:**
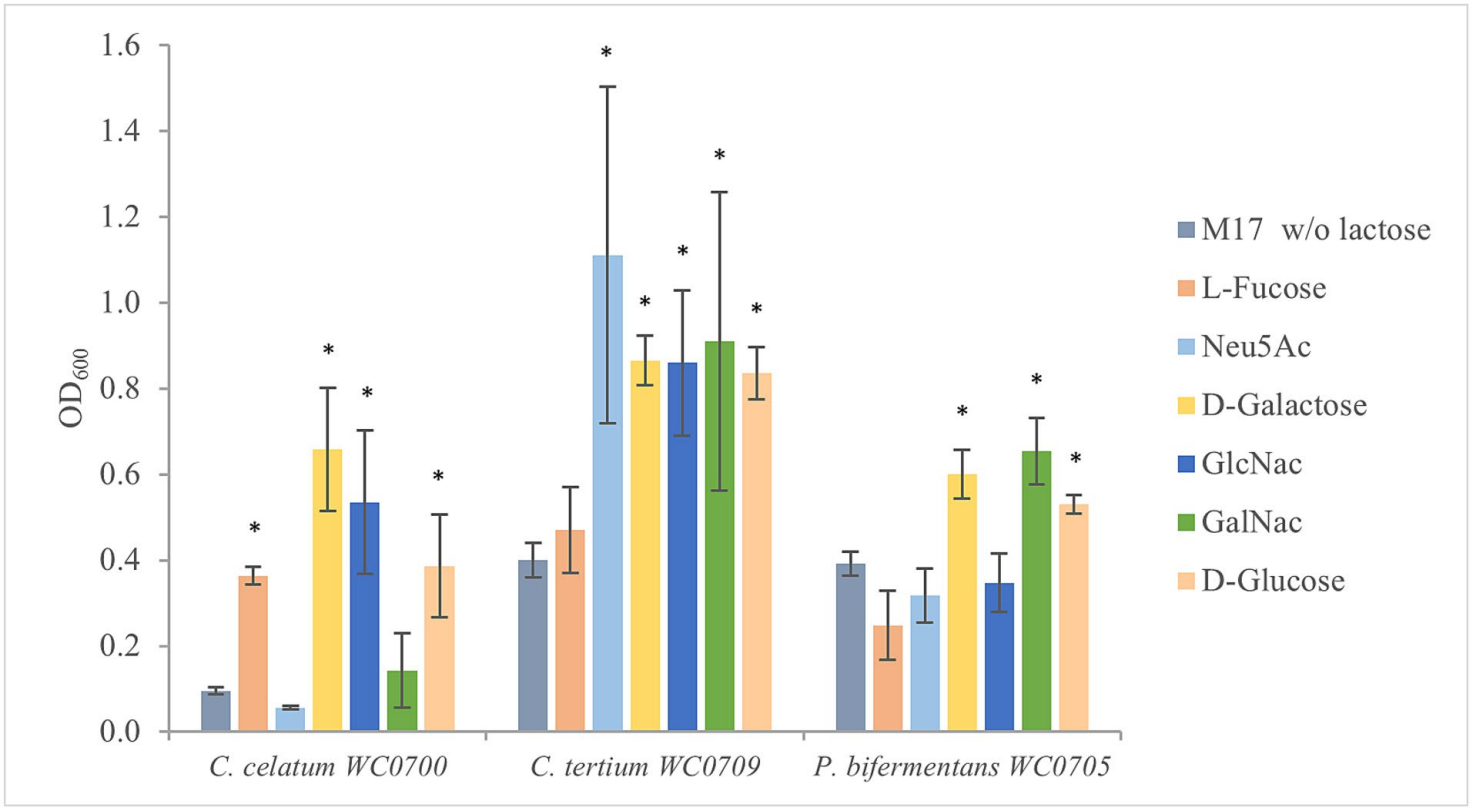
Growth of *C. celatum* WC0700, *C. tertium* WC0709, and *P. bifermentans* WC0705 on the mucin *O*-glycan monosaccharides. Bacteria were grown in M17 medium supplemented with D-glucose, D-galactose, L-fucose, Neu5Ac, GlcNAc, or GalNAc at 20 mM. M17 without carbohydrates was used as negative control. Bars show the mean OD_600_ value ± SD acquired from three independent experiments. * indicates statistical significance against the negative control (*t*-test, *p* < 0.05).

To evaluate the ability of *C. celatum* WC0700, *C. tertium* WC0709, and *P. bifermentans* WC0705 to ferment the monomers of mucin *O*-glycan, the strains were cultured in M17 containing the single monosaccharides L-fucose, Neu5Ac, Gal, GalNAc, and GlcNAc (Figure 3). *C. celatum* WC0700 grew on L-fucose, Gal, and GlcNAc. *C. tertium* WC0709 grew on Neu5Ac, Gal, GlcNAc and GalNAc. *C. celatum* WC0700 was not able to growth on GalNAc, whereas *P. bifermentans* WC0705 grew only on GalNAc and Gal.

**Figure 4:**
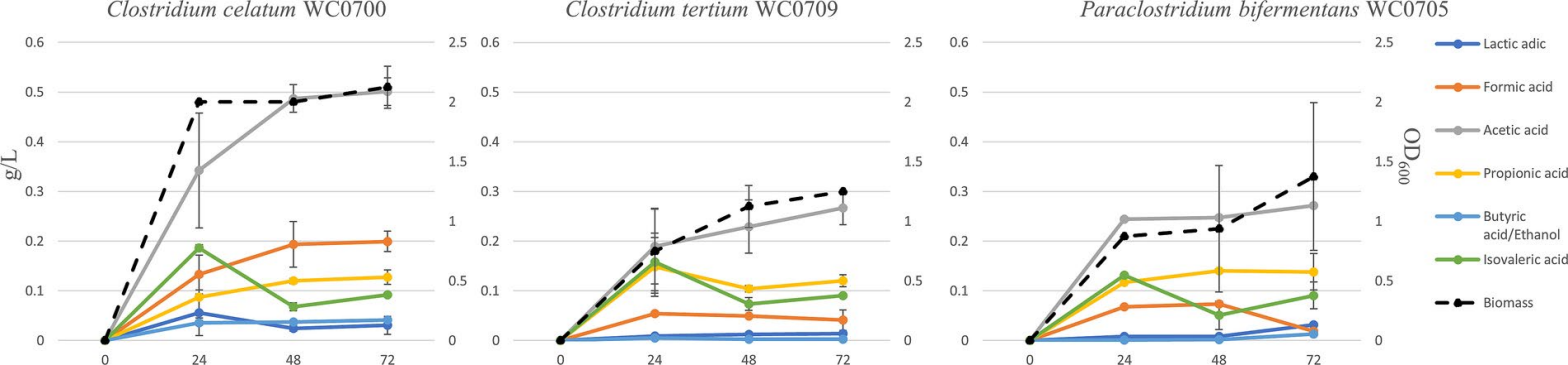
Production of biomass and metabolites in a batch process carried out on mucin as carbon source (MM broth) after 24, 48, and 72 h of incubation. Concentration of fermentation products is reported as g/L (scale on the left), while biomass as OD_600_ units (scale on the right).

To characterize mucin utilization, the strains were cultured in MM, where mucin represented the sole source of both C and N. The maximum biomass yield was generally reached after 24 h, except for *C. tertium* WC0709, which grew for 72 h (Figure 4). Mucin fermentation generated formic, acetic, propionic, butyric, isovaleric, and lactic acids, and ethanol. The abundance of each fermentation product varied among the strains, with acetic acid being generally the main primary metabolite generated. *C. celatum* WC0700 mainly produced acetic, formic, and propionic acids during growth, that reached 0.5, 0.2, and 0.1 g/L after 48 h, respectively. Growth was accompanied by the generation of isovaleric and lactic acids, both peaking at 24 h (0.2 and 0.06 g/L respectively), and by minor amounts of the other metabolites (always <0.1 g/L).

The main products of *P. bifermentans* WC0705 were acetic, propionic, formic, and isovaleric acid, which reached 0.25, 0.14, 0.07, and 0.05 g/L, respectively, after 48 h. In the cultures of *C. tertium* WC0709, acetic acid was the most abundant fermentation product, with 0.27 g/L after 72 h. Propionic and isovaleric acids were also abundantly produced in the first 24 h (0.15 and 0.16 g/L, respectively) but slightly decreased toward the end of the fermentation. In all the processes, the analysis of the headspace revealed the accumulation of CO_2_ and H_2_ as volatile fermentation products (data not shown).

The isovaleric produced in the first 24 h of fermentation decreased in all the strains, likely entering the leucine degradation pathway with energy consumption.

### 3.5 Antibiotic resistance and other phenotypes

Genetic determinants for antibiotic resistances were searched in the genome of *C. celatum* WC0700, *P. bifermentans* WC0705, and *C. tertium* WC0709. The three genomes presented several genes encoding components of eukaryotic type ABC transporters, putatively involved in multidrug efflux system (EfrA/B, MdlA, BplA) and possibly participating in antibiotic resistance mechanisms (Supplementary Spreadsheet 1). The CARD analysis revealed the presence of tetracycline resistance in *P. bifermentans* WC0705, while KEGG detected β-lactam resistance in *C. tertium* WC0709. To confirm genomic findings, MICs estimation was performed applying the microdilution method. *C. tertium* WC0709 was shown to be resistant to ampicillin (Table 6) and presented intermediate resistance to penicillin. All the strains were susceptible to chloramphenicol. *C. tertium* WC0709 and *P. bifermentans* WC0705 were resistant to tetracycline. *C. celatum* WC0700 was susceptible to all the antimicrobials tested. Interestingly, all the strains exhibited growth in presence of high concentration of gentamicin, but breakpoints were not available for defining susceptibility or resistance to this antibiotic.

**Table 6:** Antibiotic susceptibility of the *C. celatum* WC0700, *C. tertium* WC0709, and *P. bifermentans* WC0705 expressed as the minimum inhibitory concentration (MIC, mg/L).

Antibiotic	*C. celatum* WC0700	*C. tertium* WC0709	*P. bifermentans* WC0705	MIC range	Reference breakpoints	
					S	R
Ampicillin	1	8	0.125	64–0.06	≤ 4	> 8
Gentamicin	32	> 64	32	64–0.06	-	-
Chloramphenicol	2	2	4	64–0.06	≤ 8	> 8
Penicillin G	0.25	1	0.03	16–0.01	≤ 0.5	≥ 2
Tetracycline	0.06	16	16	16–0.01	≤ 4	≥ 16

Ampicillin and chloramphenicol breakpoints were defined according to EUCAST for Gram-positive anaerobes, while penicillin G and tetracycline breakpoints were defined according to Clinical and Laboratory Standards Institute. S, susceptible; R, resistant.

**Figure 5:**
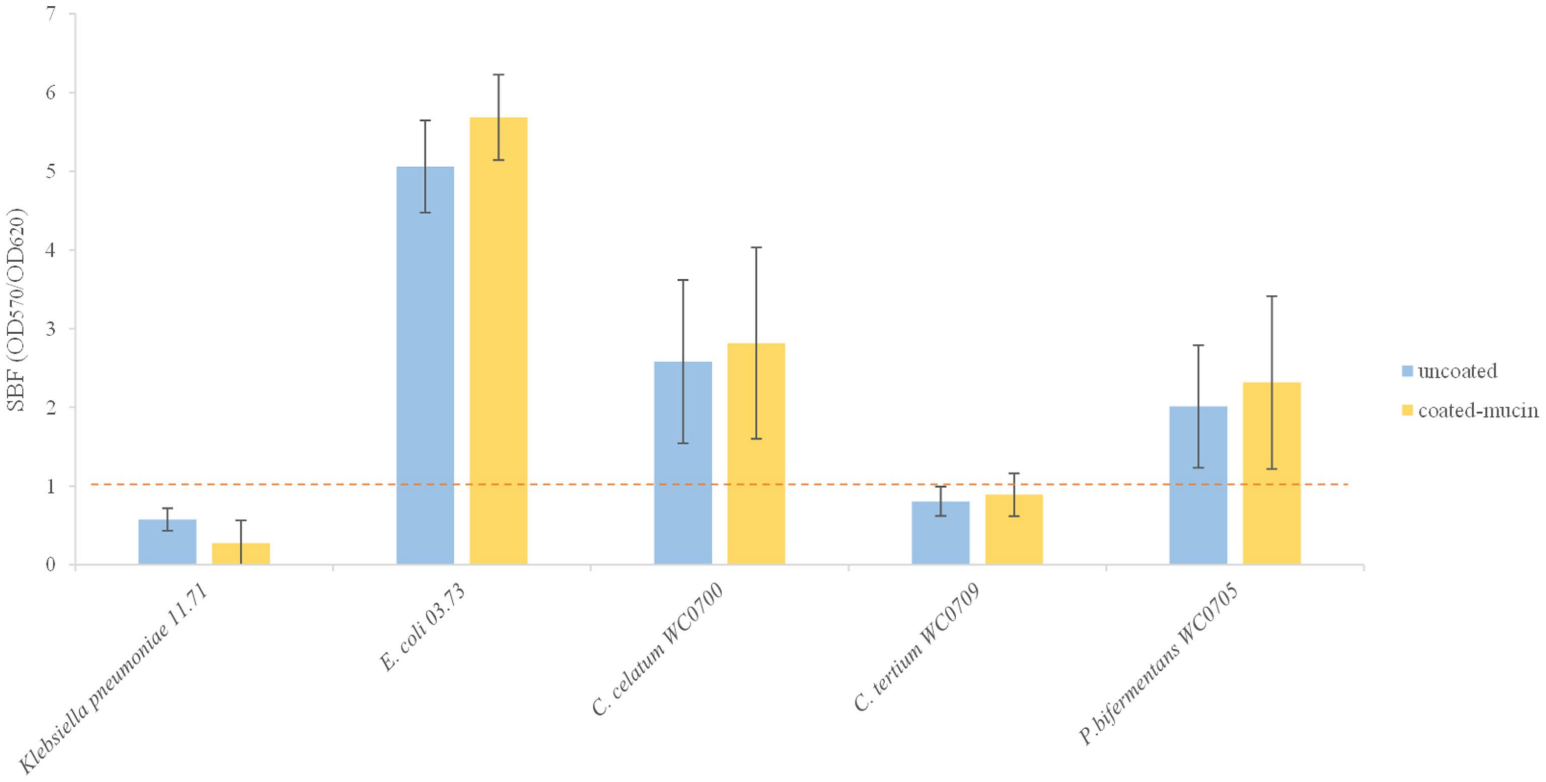
Biofilm formation in coated and un-coated mucin wells. The specific biofilm formation (SBF) is calculated as the ratio between the crystal violet absorbance at 570 nm and the culture turbidity at 620 nm, setting a threshold of 1 (red dashed line). The reported data are means ± SD of three independent experiments, each carried out in triplicate.

The three strains were tested for biofilm formation in presence and absence of mucin (Figure 5). The SBF of *C. tertium* WC0709 was under the threshold of 1, suggesting the inability to form biofilm in both conditions. Compared to the positive control *E. coli* 03.73, slight biofilm was produced by *C. celatum* WC0700 and *P. bifermentans* WC0705, with scores of 2.0 and 2.8, respectively. No statistically difference (paired samples *t*-test, *p* < 0.05) was observed in presence of mucin coating.

Survival under oxygen and high temperature exposure was assessed. Thermic and oxygen treatments, separately or combined, caused the decrease of one Log_10_ of the MPN, suggesting that the majority of the cells on the 24 h cultures were resistance spores (Supplementary Table S4).

## 4 Discussion

Most of bacteria colonizing mucus remain incompletely explored, with some exceptions such as *Akkermansia muciniphila, Bacteroides thetaiotaomicron, Allobaculum mucolyticum*, and *Ruminococcus gnavus*. In a recent work, the species *C. celatum, C. tertium*, and *P. bifermentans* were isolated from enrichment cultures of human gut microbiota on a medium containing mucin as sole carbon and nitrogen source, broadening the horizon of bacteria feeding mucins (Raimondi et al., 2021b). These three clostridia, when detected in gut microbiome of healthy subjects, are present in very low relative abundances (Candeliere et al., 2023). It is plausible that the high affinity for mucins restricts their presence to mucus, limiting the load in the luminal content, mostly reflected in microbial composition of feces.

In this new study we provide evidence that *C. celatum*, *C. tertium*, and *P. bifermentans* have a number of enzymes and transporters that allow hydrolysis of the *O*-glycan mucin chains and uptake and catabolism of the resulting monomers (Tables 2, 3; Figure 2; Supplementary Spreadsheet 1). In the intestine, mucin degradation is a cooperative affair where different microbes participate to hydrolysis of different glycosidic bonds, with release of a number of monomeric units for which each bacterium presents a specific affinity. For mucin degraders, it is not necessary to be equipped with all the enzymes involved in *O*-glycan hydrolysis, since the incomplete set of mucinolytic enzymes of each bacterium may concur to partial hydrolysis of mucin, allowing it to utilize different mucin-derived substrates. Several effectors that have a role in mucin hydrolysis, monomer uptake, and fermentation, confirmed the predicted role of *C. celatum*, *C. tertium*, and *P. bifermentans* in mucin degradation, and enlarged the repertoire of known intestinal mucin degraders. These species are able to utilize mucin in pure culture without extra C and N supplement (Figure 3), and present a wide range of glycosil hydrolases specific for mucin utilization and transport systems of the monomeric units. *C. celatum* resulted the most efficient in mucin utilization, displaying a higher number of genes involved in hydrolysis. On the other hand, *P. bifermentans* seemed less equipped in terms of enzymes and transporters, albeit it was able to grow on mucin and GalNAc as sole carbon and nitrogen source.

*In silico* and *in vitro* tests examining the utilization of mucin monomers mostly yielded comparable results. Fermentation of Gal was confirmed by *in silico* predictions, API, and fermentation tests in all the strains, despite the absence of a specific transport system in *C. tertium* WC0709. Predictions and *in vitro* experiments were also congruent for L-fucose and Neu5Ac, utilized only by *C. celatum* WC0700 and *C. tertium* WC0709, respectively. *P. bifermentans* WC0705 encoded the whole set of genes for GlcNac uptake and metabolism and resulted positive to GlcNac fermentation in API test, but it was not able to grow in MM supplemented with this monomer. A similar discrepancy was found also with GalNAc for *C. celatum* WC0700. Inconsistencies between *in silico* and *in vitro* findings may stem from limitations in annotation databases. On the other hand, divergences between predicted functions and MM growth tests may derive from the too stringent composition of the medium that could limit growth with some low affinity carbohydrates.

*Clostridium celatum*, *C. tertium*, and *P. bifermentans* can take part with other commensals in shaping the gut ecosystem and interact with the metabolism of the host intestinal epithelium. They produce a plethora of organic acids and fermentation products (Figure 4), providing essential carbon and energy sources for other gut microbes in a cross-feeding relationship (den Besten et al., 2013). On the other hand, the SCFA (acetate, propionate, butyrate, etc.) participate to activation of the immune system and to modulation of host signaling and metabolism (Lopetuso et al., 2013).

*Clostridium celatum* WC0700, *P. bifermentans* WC0705, and *C. tertium* WC0709 were unable to ferment starch and inulin, however the potential of these bacteria in hydrolyzing polysaccharides other than mucin *O*-glycan would deserve further investigation (Table 5). Nonetheless, both biochemical examinations and predictions of transporters and catabolic pathways indicated that they are capable of fermenting various simple sugars and oligosaccharides.

Although they might not serve as primary degraders, they could contribute to the complex fermentation of dietary indigestible polysaccharides, leading to the production of various organic acids (Flint et al., 2012). On the other hand, these species possess the potential to ferment amino acids, not only derived from mucin protein core, and thus they could participate in the intestinal protein breakdown metabolism, likewise several other clostridial members of the microbiome (Raimondi et al., 2021a).

Safety assessments for these strains is relevant to better understand the interaction and communication of these species with the host, and to keep opened the perspective to develop novel postbiotics, evaluating the impact on the host. Phylogenetic relationships obtained by a systematic whole genome approach based on Average Aminoacid Identity and core genome indicated that *C. celatum*, *C. tertium*, and *P. bifermentans* are ascribed to the main cluster C3 of intestinal Clostridia, characterized by a quite low GC% (Candeliere et al., 2023). According to Candeliere et al. (2023), *C. tertium* and *C. celatum* belong to the same putative genus G14, that also includes the pathogen *C. perfringens*, whereas *P. bifermentans* belongs to the strictly related genus G15. The relationship among the three species and *C. perfringens* hinted the investigation of the potential virulence. Virulence signatures were weak, with a little higher pathogenic potential for *P. bifermentans*. All the three strains encompassed the four virulence factors, homologous to those found in both *C. perfringens* and *C. difficile*, encoded by *fbpA, groEL*, and two hemolysins-encoding genes. *P. bifermentans* WC0705 shared with *C. perfringens* other virulence determinants encoded by the genes *pfoA*, *plc*, and *colA*. As a whole, the potential of virulence of *C. celatum, C. tertium*, and *P. bifermentans* is limited, according to the much higher number of genes involved in pathogenicity detected in *C. difficile* and *C. perfringens* (Supplementary Figure S3). The score of pathogenicity provided by PathogenFinder, albeit rough, suggests a higher virulence potential for *P. bifermentans*, in agreement with a higher number of genes providing an advantage in terms of survival inside the host, persistence, and infection. Interestingly, the genome of *P. bifermentans* HD0315_2, isolated from the feces of a patient with Crohn’s disease, encodes some genes for proteins involved in the infection cycle processes of *Listeria* and homologs of *C. difficile* pathogenic proteins that are absent in *P. bifermentans* WC0705 (Zhao et al., 2022). Accordingly, the score calculated by PathogenFinder for the strain *P. bifermentans* HD0315_2 (0.77, Zhao et al., 2022) is higher than WC0705 (0.69), suggesting a worsen virulence of the former strain isolated from feces of an IBD patient. Further genetic determinants for antibiotic resistances were present in the genome of *P. bifermentans* HD0315_2, possibly resulting from a more frequent use of antibiotics.

Antimicrobial resistance has a direct impact on selection of strains for probiotics or postbiotics development, chemotherapy for anaerobic infections, and horizontal transfer of resistance genes. Gut commensals frequently carry antibiotic resistance genes, providing a reservoir for pathogenic acquisition and contributing to the emergence of resistant clones of opportunistic pathogens (Lamberte and van Schaik, 2022). The isolated mucinolytic stains exhibited patterns of antibiotic susceptibility comparable to those observed in other strains of the same species from the human gut, indicating a common feature of the resident gut bacteria. *P. bifermentans* WC0705 is resistant to tetracycline, according to the presence in other strains of this species of multiple antibiotic resistance genes, including those for chloramphenicol, tetracycline, and gentamicin (Zhao et al., 2022). Also *C. tertium* WC0709 is resistant to tetracycline, consistently with the identification of tetracycline and methicillin resistance genes in the genomes of *C. perfringens* and *C. tertium* (Kiu et al., 2017). Notably, the species *C. tertium* can exhibit resistance against various other antibiotics, including cephalosporins (Leegaard et al., 2005; Sutton et al., 2017). Albeit the presence of a number of genes encoding efflux pumps and other putative antibiotic-resistance genetic determinants in *C. celatum* WC0700, *P. bifermentans* WC0705, and *C. tertium* WC0709, it is noteworthy that only a subset of antibiotic resistance genes confers resistance to clinically relevant antibiotics (Diebold et al., 2023).

Further investigation into the crosstalk between these clostridia and the host is essential to gain a comprehensive understanding of their impact on health status. Additionally, exploring their potential to develop novel postbiotics could offer valuable insights into their therapeutic applications. The challenge lying ahead is to reveal the cellular and molecular interactions between these commensals and the mucosal immune system. Experiments aimed to elucidate the regulation of immune function by *C. celatum, C. tertium*, and *P. bifermentans*, using both *in vivo* and *in vitro* models, require alive bacterial biomass. In this context, experiments conducted to assess the strains’ sensitivity to oxygen and their ability to produce resistance spores confirmed that the vegetative cells of these bacteria are highly sensitive to oxygen and utilize sporulation as a stress response strategy to survive adverse environmental conditions.

As a whole, this study advanced the knowledge of the mucin utilizing clostridial species *C. celatum*, *C. tertium*, and *P. bifermentans.* Many traits herein investigated should reassure on the safety of these species and leave ample scope for deeper investigations on the relationship with the host and for assessing if some relevant health-promoting effect could be ascribed to these SCFA producing species.

## Data availability statement

The datasets presented in this study can be found in online repositories. The names of the repository/repositories and accession number(s) can be found in the article/Supplementary material.

## Author contributions

FC: Data curation, Investigation, Methodology, Project administration, Writing – original draft. EM: Investigation, Writing – original draft. LS: Writing – original draft, Investigation, Validation. AA: Investigation, Validation, Writing – original draft, Methodology. SR: Investigation, Methodology, Validation, Writing – review & editing. MR: Conceptualization, Funding acquisition, Writing – original draft, Writing – review & editing.

## Footnotes

^1^https://www.eucast.org/clinical_breakpoints/, version 11.0.

^2^https://clsi.org/meetings/microbiology/, M100-Ed31:2021.

^3^usegalaxy.eu/

^4^https://www.dsmz.de/services/online-tools/genome-to-genome-distancecalculator-Ggdc

^5^https://www.genome.jp/kegg/module.html
